# Understanding the unintended consequences of public health policies: the views of policymakers and evaluators

**DOI:** 10.1186/s12889-019-7389-6

**Published:** 2019-08-06

**Authors:** Kathryn Oliver, Theo Lorenc, Jane Tinkler, Chris Bonell

**Affiliations:** 1 Department of Public Health, Environments and Society, London School of Hygiene and Tropical Medicine, 15-17 Tavistock Place, London, WC1H 9SH UK; 2 Centre for Reviews and Dissemination, University of York, York, YO10 5DD UK; 3 Department of Sociology, Social Policy and Criminology, University of Stirling, London, UK

## Abstract

**Background:**

Public health policies sometimes have unexpected effects. Understanding how policies and interventions lead to outcomes is essential if policymakers and researchers are to intervene effectively and reduce harmful and other unintended consequences (UCs) of their actions. Yet, evaluating complex mechanisms and outcomes is challenging, even before considering how to predict assess and understand outcomes and UCs when interventions are scaled up. We aimed to explore with UK policymakers why some policies have UCs, and how researchers and policymakers should respond.

**Methods:**

We convened a one-day workshop with 14 people involved in developing, implementing or evaluating social and public health policies, and/or evaluating possible unintended effects. This included senior evaluators, policymakers from government and associated agencies, and researchers, covering policy domains from public health, social policy, poverty, and international development.

**Results:**

Policymakers suggested UCs happen for a range of reasons: poor policy design, unclear articulation of policy mechanisms or goals, or unclear or inappropriate evidence use, including evaluation techniques. While not always avoidable, it was felt that UCs could be partially mitigated by better use of theory and evidence, better involvement of stakeholders in concurrent design and evaluation of policies, and appropriate evaluation systems.

**Conclusions:**

UCs can be used to explore the mechanisms underpinning social change caused by public health policies. Articulating these mechanisms is essential for truly evidence-informed decision-making, to enable informed debate about policy options, and to develop evaluation techniques. Future work includes trying to develop a holistic stakeholder-led evaluation process.

## Background

To implement effective policies and interventions in fields such as public health or social policy, decision-makers need to consider what works, for whom, and under what circumstances [1, 2]. Questioning how interventions attain their stated goals is the heart of evidence-informed decision-making (EIDM), and is the main focus of methods to evaluate interventions. Less attention has been paid to the unintended consequences (UCs) of interventions, that is, the ways in which interventions may have impacts – either positive ‘spillover’ effects or negative harms – not planned by those implementing them.

Adverse effects have always been a part of clinical research. Understanding the side effects of drugs and procedures is as important as their clinical effectiveness, when deciding whether to use them in treatment. Clinical researchers are required to report and monitor adverse effects, interventions go through multiple rounds of testing to explore possible effects, and the modes of action of clinical interventions are usually well-articulated. In public health, however, it is harder to connect changes in social outcomes to specific interventions, and even harder to articulate mechanisms underpinning these changes [3]. For example, policy interventions which change the built environment will affect people differently according to where they live, their use of the public space, their age and sex, amongst other factors. Even aside from this, complex social or policy interventions may have unexpected impacts at a population level, for reasons which are hard to predict in advance. Finally, social and policy interventions are not regulated and monitored in the same way, meaning UCs could be harder to identify.

Reducing harm, and gaining a more complete understanding of why policies and interventions have the effects they do, can help policymakers to intervene more successfully in social systems, as well as informing researchers about the mechanisms underlying social change [4]. Yet, our current understanding of how policies are made suggests that there is limited testing of policies [5], that the potential for evidence to be used is not always maximised [6–9], and that the underlying models and theories of policies are not always made explicit [10–13]. These characteristics of the policy process may lead to UCs, and offer potential pathways to their alleviation.

Therefore, this project was designed to enable us to learn from stakeholders’ views about the unintended consequences of policies, to seek their advice about important research topics in this area and key examples to explore, and to identify potential avenues for future enquiry.

## Aims

Building on our previous work examining adverse effects of public health policies [3], this project aimed to gather stakeholder perspectives on how UCs of policies and interventions arise in order to develop ideas for future research into unintended effects caused by public health policies and interventions. This paper focuses on why unintended consequences may arise, and how researchers and policymakers can attempt to respond. Another paper focusing particularly on issues of evaluation has already been published [14].

## Methods

We sought to develop our understanding of how UCs are perceived by policymakers and researchers by holding a one-day workshop with senior UK policymakers and researchers with interests in public health, social policy, development, poverty and evaluation (*n* = 14). We used a focus group design (following Petticrew/Whitehead [15, 16]) to allow enough time for discussions to evolve naturally, enabling us to capture the complexities of the policy and research issues associated with unintended consequences. While they cannot capture data with the same granularity as interviews, focus groups are useful to bring together a range of stakeholders, and to enable discussion between those with different perspectives [17]. This discussion can, as was the case with our workshop, allow participants to build on and engage with each other’s responses. It was important to us to achieve this as we anticipated that collecting data about UCs would be challenging, that people may not always be aware of or label their own experiences as to do with unintended consequences without prompting, and that hearing others talk may spark new ideas and connections. This fitted with our aims to explore a range of perspectives, rather than generate rich, exhaustive accounts of unintended consequences about particular cases.

We contacted 44 senior officials at key UK1 organisations by email, with a reminder within a fortnight (e.g. Public Health England (PHE), Department of Health (DH), Food Standards Agency (FSA)) and invited a representative to attend. We identified the key public health and policy organisations through consultation with policy colleagues (e.g. at PHE) and through examining key organisational governance structures. We aimed purposively to recruit a range of policy colleagues working at different levels, in different roles (e.g. evaluation, policy development, implementation, research and strategy) in different areas, to allow us to identify key issues for research into unintended consequences and to seek advice about how to explore these at all levels.

Of the 44, 12 originally accepted but then pulled out, 8 declined and 8 never answered (mainly local public health officials). Two attendees were unable to come on the day, leaving 14 participants. They included heads of department, senior strategies, academics, and evaluation leads at institutions such as the Government Evaluation Unit, NatCen and the Office for National Statistics (Table 1).

**Table 1:** Participant characteristics (assessed by authors)

	Area of work	Employer type	Experience
1	Social policy	Third sector	Senior
2	Drugs policy	Government	Senior
3	Evaluation	Independent public body	Mid-level
4	Public health	University	Senior
5	Evaluation	Independent research institute	Mid-level
6	Evaluation	Independent public body	Mid-level
7	Public health	Independent public body	Mid-level
8	Public health	University	Senior
9	Public health	Government	Mid-level
10	Evaluation	Third sector	Mid-level
11	Evaluation	Private industry	Mid-level
12	Health policy	Government	Mid-level
13	Public health	Government	Senior
14	Public policy	Independent public body	Senior

We conducted three in-depth sessions, facilitated by authors, exploring the following questions: (1) Why do policies and interventions have UCs, (2) how to manage UCs, and (3) evaluating UCs. Each session included a brief presentation from a facilitator presenting the main evidence in the area, some examples of harmful interventions, and the key questions for discussion. These were followed by small group discussions, feedback to the larger group and refinement of concepts and ideas. The facilitators introduced examples to work through (including alcohol prohibition [18], juvenile recidivism deterrence [19] and school vouchers [20] but participants were encouraged (in advance, and on the day) to provide their own examples. Within each session, we facilitated discussion about the broader questions concerning UCs, such as eliciting examples, and thinking through the political, logistic and ethical ramifications.

At all times, the facilitators observed and took part in the discussions, asking for clarifications and summarising discussions regularly. We took notes separately about each discussion, and collected data verbatim where possible (following Petticrew and Whitehead [15, 16]). At the end of the day we shared these reports amongst the facilitators. To organise these, KO read each set of notes and identified common themes (e.g. ‘challenges’, ‘examples’) through a close reading and annotation of the text, using word processing software. The themes arose inductively from the reports and in discussion with the other facilitators. These were shared with the other facilitators, and the notes were re-examined by each against these themes to ensure these were coherent. No a priori coding scheme was used, as we wanted the findings to be led by themes identified by participants. KO collated each set of notes into a single document, organising the data under each theme. Each theme was then reported, prioritising the participants’ interpretations. Each theme was shared with JT and TL, and discussed in depth to ensure there were no missing themes, and that the interpretation made by KO was correct. We did not attempt to critically interpret these themes, or to engage in any theory-driven analysis, Rather, we present these data as offered by participants, to allow readers the sense of the discussions on the day, and to reflect the stakeholders’ perspectives with as much integrity as possible. This method was useful to elicit thoughts and responses, which was our aim, but may not be appropriate for other aims, such as the crafting of more detailed theory.

At times, participants mentioned specific examples of policies or research publications. To aid the reader, the authors have attached relevant references describing the policy or intervention in question.

Chatham House rules were agreed, meaning participants were free to use information received, but neither the identity or affiliation of speakers may be revealed. Thus all participants could feel confident of speaking freely and honestly about potentially difficult issues such as policy failures or mishandling. We also agreed with participants that no comments would be attributable to individual speakers, which is why we have not identified the posts or roles of participants.

Participants gave their consent for verbatim and summary notes to be used in the preparation of this and other publications. The results below draw directly on the thematic notes, which are verbatim reports as far as possible. The characterisations and inferences made are drawn by participants, not the authors.

## Results

We captured views about how policymakers and researchers define, describe, anticipate and plan to evaluate UCs. Below, we summarise the key points under each theme identified: the ‘nature of UCs’; the ‘causes of UCs’ (subthemes: policy design, unclear policy goals, policy implementation / evaluation); ‘evidence use’; and ‘Responding to UCs’.

There were significant commonalities between the participants’ views, which are summarised below. We did not attempt to achieve thematic or theory saturation, as this workshop aimed to inform our thinking about the potential to investigate UCs, rather than to assert a definitive account. The results are therefore still informative.

### The nature of unintended consequences

Broadly, participants agreed politicians and interventionists are motivated by the desire to improve social outcomes, and believe that their actions will work. It was accepted that policymakers knew that sometimes not all policies worked for all, even harming some. Politically, this means that discussion of UCs, let alone evaluation of them, was challenging. Admitting to uncertainty was difficult for policymakers who often felt they were fighting to maintain a position.

Participants distinguished between UCs caused by counterproductive policy (which has the opposite effect to that intended), ineffective policy (no effect), and those which were by-products, or out of scope (affected other outcomes or populations than those intended). For example, Scared Straight was counterproductive [19], NHS reorganisation(s) was ineffective, and the smoking ban had unexpected (positive) effects on short-term cardiac deaths [21], where only long-term outcomes had been envisaged. This is a similar division of harms as proposed by Bonell et al., with counterproductive policies analogous to paradoxical effects, and the out-of-scope to harmful externalities [4], but additionally identifies ‘null results’ as an UC.

Participants felt some harmful UCs were acceptable, but others required immediate attention. For example, cycling to school schemes benefitted children’s health, but could also lead to increased injuries and emergency hospital visits. Balancing the positive and negative effects was a task for decision-makers, but it was noted that the most disenfranchised often bear the brunt of UCs, although their distribution is never fully predictable in advance.

Most participants felt that UCs were very common – with some arguing that *any* sufficiently complex intervention will have some UCs – but that data on them were not usually systematically collected. They also observed that what counts as a UC is not necessarily an objective fact but rather a matter of perspective: consequences may be unintended or unexpected by some but not by others. This led to a discussion about how sometimes policymakers were ‘outsiders’ to social situations, meaning they were not well-placed to design interventions in that they had little experience of implementing policies.

### The causes of unintended consequences

Participants felt that there were a range of reasons and multiple causes for UCs, not all of which were under our control. Some cautioned against assuming that ‘better’ policy design would lead to reduced potential for negative impacts, and that it was never really possible to know in detail how a policy would affect all groups. For those, the question was not ‘how to avoid’ UCs, but rather ‘what’s the best we can do as policymakers?’ Endorsing realistic assessment of insight into the consequences of complex policies, participants cautioned against the ‘illusion of control’ by trying to exhaustively identify all possible UCs.

Nevertheless, participants stressed the importance of learning from UCs, and discussed a number of factors which may contribute to UCs:

#### Policy design

Some participants felt that policies were not always designed sufficiently well to achieve their intended goals. Policymakers are trained to develop policy using rational-actor models, which participants felt were not always, perhaps never, appropriate. Policy silos meant underlying assumptions were not challenged. It was widely acknowledged that blunt policy tools have multiple effects, and thus it is not always easy to carry out a ‘surgical strike’ to change one outcome – yet silos tended to reinforce this linear way of thinking about outcomes, populations and contexts which are in reality complex.

Some felt that increased testing of policies would help to alleviate this problem. Unlike clinical trials [22], social policies do not undergo several rounds of testing and refinement. How seriously one needs to take UCs partly depends on their place in the policy ‘life-cycle’, but existing mechanisms of evaluation and feedback may not clearly distinguish ‘teething problems’ from more lasting UCs. This means that it is less clear what the effects will be, on whom, and by what mechanisms they will come about. Different population subgroups will respond in different ways – so population-wide theories of change may not give accurate predictions of impact.

Conversely, others felt that policymakers were good at, and received training in, policy design but not in implementation or evaluation, where UCs were also found.

#### Unclear policy goals

Relatedly UCs sometimes came about because of the way policy goals were articulated, in that these were not always well defined, so a policy may succeed on its own terms but still have UCs.

Identifying the goal or goals of a policy may not be a straightforward matter. Participants discussed how the goals of policies are often intentionally ambiguous, and depend on the context and on the audience being addressed. (This also has implications for our understanding of evaluation – as one participant put it, evaluation is necessarily valuation.) Also, policy actors may have to emphasise a narrow subset of their aims for reasons of acceptability or political strategy. For example, the smoking ban in the UK was initially framed as a question of employees’ rights to avoid harms from passive smoking, rather than as an intervention to reduce smoking rates and the associated harms to people who smoke.

#### Policy implementation and evaluation

It was recognised that a policy is not a discrete event. Some participants questioned the habit of referring to policies or interventions as well-defined entities, suggesting that the policy process is in reality more complex and messy than this. Much depends on details of implementation, so talking about the UCs ‘of’ a policy elides what happens between the strategic policy idea and the policy as implemented or ‘enacted’ in real-world contexts.

Local and appropriate governance systems were not always considered in the roll-out of policies, leading to UCs. For instance, child benefit in the UK was always given to women, until the consolidation of benefits under Universal Credit [23–25]. This change has led to economic power being transferred to men, disempowering women and children [26].

Similarly, interventions that work well at a local level may be rolled out at a broader scale without appropriate testing and implementation [27]. National policies like People’s Health Trust [28] or NHS new service models [29] demand integration of care, but provide no definition of integration [27]). This can lead to a mismatch between systems: e.g. health and social care that are assumed to be working in tandem, but where in fact cuts to health lead to greater strain on social care.

In addition, policies achieve political momentum, so that once a policy has been put in place, especially if it is costly or high-profile, it can be very hard to change, leading to negative effects. Participants suggested that in practice evidence of UCs is held to a higher standard of proof than evidence of positive impacts. Some participants also pointed to the long timescales involved in evaluation, such that by the time evidence is reported policy thinking has moved on and the evidence is no longer relevant – which is of course relevant beyond the analysis of UCs.

### Evidence use

Cutting across these themes was a discussion about evidence use: in developing policies, regarding the involvement of stakeholders, and in evaluating polices.

Some participants felt that UCs indicated that a policy problem had not been well posed in the first place, or not based on an evidence-informed theory. Policy-makers needed to consider the intervention logic, and how it might interact with the implementation context. As a possible symptom of this, policies often did not reflect expected variations in behaviour of service users or the broader public. As outsiders to the implementation context, policymakers often did not draw on user views which could provide information relevant to how and when the policy will play out. Participants discussed involving experts and other stakeholders in the policy process. There was a feeling that being more thoughtful about how to select salient experts, and how to consult, value and include multiple voices would help to avoid UCs.

Evidence used was mostly discussed with reference to evaluation methods. UCs were often connected with the selection of outcomes for the evaluation. As discussed above, the question of policy goals always has a political dimension, so the choice of appropriate outcome measures may be a politically motivated process. For example, the Scared Straight evaluation preferred by proponents of the policy shows raised awareness of prison immediately following the visit [19, 30, 31], which they argue demonstrated effectiveness. It thus becomes possible to tell different narratives form the same policy. Sure Start is talked about both as a success [32, 33] and a failure [34, 35], according to whether one measured social exclusion / participation, or educational attainment.

### Responding to unintended consequences

Participants were split over whether it was possible to predict or identify UCs. Some felt that this was an unachievable goal in most cases, while others pointed to concrete ways in which uncertainty could be addressed: involving consumers, service users and other stakeholders; testing and piloting interventions; designing ‘nested’ interventions which contain components to mitigate anticipated UCS; and conducting formative research to better understand the context. Understanding the drivers of policies could also help observers to understand the mechanisms by which policies are likely to lead to effects, although only in cases where clear policy goals are agreed and articulated.

Participants discussed how challenging it can be to identify and present UCs, particularly negative effects. Politicians prefer narratives of success, and are under pressure not to admit to ‘U-turns’. In fact, they will often maintain publicly that a policy is being continued when it isn’t. Admitting UCS is equivalent to admitting failure and this can only be done with political support. Public opinion and scrutiny of politicians can lead to positive spin rather than reflective practice.

At times, policymakers may find it easier to respond to evidence of UCs if they are not particularly wedded to a policy. At other times, they may react with denial / anger – it is hard to admit something doesn’t work, especially if it is a core ideological belief held by the proponent. This can also apply to experts advising on policies backed by long-running research projects. In addition, poor evaluation practices make it easy to dismiss reports of UCs.

There are political and technical challenges to evaluation, and ethical and moral issues to consider. Yet, avoiding UCs would improve the effectiveness of policymaking, reduce waste, and allow more focused interventions. Participants discussed a number of ways to try and address this need:

First, policymakers could recognise that a suite of interventions is usually required to achieve sustainable change in an outcome. Accepting that trade-offs need to be made, and communicating these is essential to a transparent system.

Secondly, involve the appropriate stakeholders and attempt to achieve a conversation about the overall story of an intervention or a policy – leading to a revision of the underlying theory. This story should list the consequences, setting out the theory of chance with stakeholders, considering the whole life-cycle of the policy. The theory of change should be revisited throughout the policy, although in practice it may be hard to be completely adaptive. If the policy implementation is phased (e.g. auto enrolment) then this allows for adaptation as it progresses. Key assumptions in the theory of change could be tested, and design and evaluation run concurrently.

Finally, accept that while it may not always fit the political discourse to admit it, if policymakers are made aware of UCs, they may address them behind the scenes, by running parallel policy development processes, for instance.

## Discussion

### Key findings

Unintended consequences are common and hard to predict or evaluate, and can arise through all parts of the policy process. They may come about through ineffective (null effect), counterproductive (paradoxical effect), or other policy mechanism (harmful externalities). They are rarely evaluated systematically [14, 36, 37], and there are major technical and political challenges to doing so - but substantive, ethical and moral reasons why we should [3, 4].

Asking policymakers and researchers to consider UCs provides a rich resource to think about the mechanisms by which we think policies work: in other words, to articulate sets of hypotheses about how social change happens. These workshops showed policymakers and researchers grappling with the complex reality of attempting to intervene in ever-changing social systems. In line with recent calls to consider policy a complex adaptive system [38], we observed very sophisticated reasoning about how to manage uncertainty [9]. This concurs with presentations of policymakers as attempting to negotiate wicked problems and solutions [39, 40]. However, there remain important differences in how participants discussed the policy process, and the ways in which UCs may be created and identified, and our understanding of policy as represented in the literature. For example, participants often referred to heuristics such as the policy cycle, which many commentators regard as an unhelpful device for analysing policy [41]. There were also several discussions about the relative strength of different forms of evidence, the need for rational decision-making in response to research evidence, and references to ‘upskilling’ policymakers, all of which offer a far more normative view of the policy process than ordinarily found in the policy studies literature (see, e.g. [12, 42–46]).

Evidence use was a major theme. ‘Evidence-informed policy’ has too often referred to the evaluation of policies [47, 48]; and some participants suggested that there was a role for increased testing and piloting of policies prior to implementation, in order to identify unintended effects. However, without being able to discuss specific scenarios, we were not able to explore the logistics or practicalities of this approach, although we recognise this is a key area for future research (see, for example [5]). We suggest there is an equally important role in the development of theories of change, mechanisms, or logic models (all analogous terms). Yet, this role is underemphasised by commentators on evidence-based policy [49, 50]. Rather, a better understanding of the various interactions between evidence production and use is required, with attention to systems, processes and actors, as well as outcomes.

Many participants emphasised the ambiguity and contingency of the policy process, which means that linear narratives connecting a single intervention to a limited set of fixed outcomes are idealisations at best. Moreover, the identification of policies and goals is itself always political in nature, and implicitly prioritises the interests of certain actors or groups over others; and policy itself was described as multiple with multiple goals. This has long been recognised in the literature on policy studies [51–55], but its implications for the evaluation of the effectiveness of policies, and the utilisation of the resulting evidence, have not always been recognised. At several times in the discussion, participants discussed artefacts of the policy process (e.g. the use of ‘blunt policy tools’, the ‘policy cycle’) or characteristics of the policy actors (e.g. preferring ‘rational actor’ models) which were surprising to us, as from a policy studies perspective these represent rather normative views of the policy process [55]. Further research would be required to interrogate how different participants conceptualised or operationalised these ideas, how widely they were shared, and the currency they hold in the practice of policy.

Running through all the discussions was a theme about public engagement. Listening to the right voices, at the right time, may not be a panacea for unintended consequences, but better use of public deliberation may make policy more effective, and more predictable [42, 56]. Also, balancing the positive and possible negative effects of policies and interventions implies a deliberation takes place by decision-makers. It would be interesting to know how open and explicit that deliberation is, and whether it is inclusive of relevant stakeholders – and indeed whether deliberative approaches do lead to better policymaking practices [57, 58].

As some participants noted, unintended consequences may fall most heavily on the most disenfranchised. Thus, thinking carefully about who is likely to be affected by policies is a question of equity, and one which can be addressed through mindful stakeholder engagement.

The aim of these workshops was not to provide rich, in-depth accounts of the perspectives of policymakers, or to generate evaluations of specific unintended consequences. Neither is the methodology a one-size fits-all approach, but designed here to elicit frank and open responses in response to particular provocations. Rather, we aimed to report perspectives of stakeholders, without relating these to existing work on policy theory or evaluation, in order to give a clear picture of how policymakers and researchers view the complexity of the task facing those researching and managing UCs. Therefore, we have not attempted to critique particular statements, or to impose our own ontological views about evidence production and use on these findings. Rather, we hope that these results offer a set of questions for future researchers, building on these findings. In particular, we believe that the following would be fruitful avenues for discussion:

1. An exploration of the implicit models of policymaking which are offered by these perspectives about unintended consequences.
2. The mechanisms which are implicitly or explicitly used to develop or evaluate policies, or which conceptually underpin policies.
3. The role of evidence in supporting these mechanisms, and particularly the potential for coproducing mechanisms to inform policy development, evaluation and implementation
4. The importance of identifying unintended consequences for public health in particular, which can affect entire populations
5. The role of evaluation, monitoring and reporting, and governance of public health policies in identifying and mitigating unintended consequences; and
6. How best to adapt existing evaluation frameworks to enable a better understanding of unintended consequences in public health

We also believe there would be significant value in systematically identifying all policies in public health which have had unintended or harmful effects, to begin the work of understanding and avoiding this phenomenon. We believe that public health policy interventions, particularly those addressing social or environmental determinants of health, need to be seen within a broader understanding of the policy process. Hence, we aimed to access perspectives from a range of fields, on the assumption (which we think is borne out by the findings) that participants’ views and experiences would have similarities across sectors.

### Implications

Finally, we note that while all participants accepted the idea that policies and interventions may have unexpected effects, this is rarely taken into account by research or evaluation funding. Honest policymaking requires a holistic understanding of the ways in which policies play out. This should include equal humility from researchers and commissioners about the ways in which we do not understand, or fail to predict, the impacts of our interventions on social systems.

### Limitations

This workshop was relatively limited in size, and we did not have access to complete transcripts. We also conducted the analysis inductively, aiming to privilege participants’ accounts and reports, rather than our own interpretation. This has meant that we have at times conducted a theoretically naïve analysis, and we acknowledge that this may have biased results. However, we intended to produce a set of questions for future investigation rather than produce a rich account of policymakers’ accounts – work which we believe is important, and necessary, but we are not able to make those kinds of claims given the data available to us. Instead, we delineate a novel field of enquiry for public health research.

## Conclusions

Unintended consequences of policies and interventions are occasionally, but not systematically reported. Little is known about how they arise, if they fall into categories, or how to evaluate and respond to them. Thinking about unintended consequences of policies can help us to learn about how policies and interventions play out, and the actual mechanisms leading to social change. Our study suggests that developing better theories about how policies will work requires input from people who will be affected by the policy, and by those involved in developing and implementing it.

## Data Availability

Data collected is not available for sharing, as the workshops were conducted under Chatham House rules in order to protect participants.

## Abbreviations

DH: Department of Health
EIDM: Evidence-informed decision-making
FSA: Food Standards Agency
PHE: Public Health England
UCs: Unintended consequences
UK: United Kingdom
